# Social workers’ perceptions of assessing the parental capacity of parents with intellectual disabilities in child protection investigations

**DOI:** 10.1177/17446295221113717

**Published:** 2022-07-12

**Authors:** Jerry Norlin, Eva Randell

**Affiliations:** 1 School of Health and Welfare, 3317 Dalarna University, Falun, Sweden

**Keywords:** Child protection investigations, Intellectual disability, Parental capacity assessments, Qualitative study, Social workers

## Abstract

Parental capacity is one of the main aspects assessed by social workers as part of child protection investigations. The aim of this study is to explore the social workers' perceptions of assessing the parental capacity of parents with intellectual disabilities in child protection investigations. Four focus group interviews were conducted with twelve social workers in May-October 2021. Data were analysed using an inductive, conventional qualitative content analysis. One overarching theme, “Parental capacity in relation to the child's needs was assessed, not disability of parents” was created. Three main categories and ten sub-categories were identified exploring the social workers' perceptions of assessing parental capacity. The study shows that social workers perceive the assessment of parental capacity in parents with intellectual disabilities as demanding and complex, in which the assessment of what is good enough is perceived as the most difficult task.

## Background

### Introduction

When social workers in child protection services evaluate how well a parent can care for their child, they are determining what is known as parental capacity. Previous research in the field of social work has found that assessments of parental capacity are viewed as particularly complex when parents have cognitive difficulties such as learning disabilities or intellectual disabilities (Lewis et al., 2015; Ward et al., 2015). This study deals with social workers’ assessments of parental capacity in parents with intellectual disabilities in Sweden.

### Social workers’ assessments of the parental capacity

The responsibilities of the social services in Sweden are regulated by the Social Services Act. This legislation considers the needs of both children and parents as part of a general family-oriented discourse in social welfare. This approach includes working with the family and the wider network surrounding the child. Sweden has also agreed a national strategy with the goal of offering support to all parents regardless of problems or risks and targeted support to parents within certain risk groups (Ministry of Health and Social Affairs, 2018).

One of the main tasks of social workers in the child protection services is to assess and determine what help and support is needed in cases where children are at risk of neglect, or if the children’s support needs cannot be met by their parents. Thus one of the social workers’ main objectives in the child protection investigations is to assess the parental capacity of the parent (Aunos and Pacheco, 2021). The term “parental capacity” should not be confused with the term “parental ability”, as the former is defined as the ability to parent in a good enough manner for a long term, whereas the latter is defined as being able to parent under specific circumstances for a short period of time (Conley, 2003). Previous studies show that assessing parental capacity is perceived as a demanding task by social workers and may be especially challenging when social workers meet parents who have intellectual difficulties (Lewis et al., 2015; Ward et al., 2015).

Definitions of intellectual disability vary. Systematic reviews based on meta-analysis estimates of the prevalence of disability within national populations report great heterogeneity in population characteristics and settings (Maulik et al., 2011; McKenzie et al., 2016). Some studies of prevalence have shown that approximately 1% of the global population have intellectual disabilities (Maulik et al., 2011), while others show that it might be slightly less than 1% (McKenzie et al., 2016). One study from Ireland highlights the difficulties of obtaining accurate statistics on the prevalence of intellectual disabilities using national register records, as population samples might vary from the actual prevalence of intellectual disabilities in a country (McConkey et al., 2019). In Sweden, the prevalence of intellectual disability in the population is not registered by the authorities, but in a sample of high school students ranging from 16-20 years old, it is suggested that approximately 2.4% of the total cohort of students in the same age range have an intellectual disability (Arvidsson et al., 2016).

In 2006, the child protection services in Sweden implemented a new procedure for the management, implementation and following-up of the social services for children and youth. The procedure is called BBIC and literally translated means “*Children’s needs in focus*” (National Board of Health and Welfare, 2018). The procedure is established by the Swedish National Board of Health and Welfare as a means to make the way the national child protection services work more consistent and systematic. The procedure is organised using a framework consisting of three main components: fundamental principles, documentation, and the BBIC triangle (National Board of Health and Welfare, 2018; Matscheck and Berg Eklundh, 2015). The BBIC triangle represents the three main areas of a child’s needs: the child’s development, the family and environment and the parent’s capacity. Each of these areas is in turn linked to risk and protective factors in the child’s direct proximity (Matscheck and Berg Eklundh, 2015; National Board of Health and Welfare, 2018). Another tool used in conjunction with the BBIC procedure in the assessment of risk in child protection investigations is the Signs of Safety approach (Edwards et al., 1999). The tool is based on a participatory approach and involves using different planning protocols and risk assessment tools in collaboration between social workers and families to map in worries, risks and strengths surrounding the families’ ability to cope (Turnell and Murphy, 2014). With Signs of Safety conclusions based on risk assessments and safety plans developed in collaboration with the families are based on the notion that parents are experts regarding their own situation (Baginsky et al., 2021; Caffrey and Browne, 2022; Edwards et al., 1999).

Risk and protective factors are described in research as phenomena such as behaviours, situations, circumstances, characteristics, relationships or traits that can increase or decrease the probability of an incident or an outcome occurring (Andershed and Andershed, 2015). Researchers have found a significant correlation between the accumulated risk factors during childhood and future problematic outcomes in adolescence (Ahonen et al., 2015; Appleyard et al., 2005; Loeber et al., 2009). By investigating how each factor corresponds to the present situation during a child protection investigation, the social workers can identify both risk and protective factors in the parental capacity. After a balanced assessment the social workers are authorised to determine what interventions might reduce risk factors or strengthen protective factors in each specific case (Andershed and Andershed, 2015; National Board of Health and Welfare, 2018).

### Parental capacity and intellectual disabilities

Research on the children in child protection services shows that children of parents with intellectual disabilities are overrepresented in cases where authorities have made the assessment that a child needs placement outside of their original family (Llewellyn et al., 2003; McConnell et al., 2011; Tøssebro et al., 2017). Research also shows that children of parents with cognitive limitations are more likely to be placed in out-of-home care (Booth et al., 2006; Emerson et al., 2005; Tøssebro et al., 2017).

The component “Parent’s capacity” in the BBIC triangle is made up of four main areas, which in turn include twelve sub-areas that the social worker can focus on when making their assessments of parental capacity. Each sub-area contains descriptions of both risk and protective factors linked to the parents’ parental abilities (National Board of Health and Welfare, 2018). These main areas are: basic care, stimulation and guidance, emotional accessibility and safety (National Board of Health and Welfare, 2018). These areas are also acknowledged as important in research on what factors undermine a parent’s capability to parent (Ward et al., 2015). Research on the parental capacity of parents with intellectual disabilities has found that parents with intellectual disabilities have a higher risk of struggling within multiple areas regarding the needs and care of their child (Emerson and Brigham, 2013; Feldman et al., 2012; Lindberg et al., 2017; McGaw et al., 2010; Sidebotham and Heron, 2006).

In the UN Convention of the Rights of Persons with Disabilities, article 23 states that in matters relating to starting families and the right to have children, persons with disabilities must be ensured the same rights and support as others to be able to pursue a pregnancy and rear children (United Nations, 2006). Even though different aspects of the parent’s capacity in the BBIC model can be viewed as guidelines on what to consider when assessing the parental capacity of parents with intellectual disabilities, it is still important to satisfy both the rights of the parents and the children. The social workers who are conducting the assessments need to be trained to evaluate the risk and protective factors associated with the parents (Andershed and Andershed, 2015), a process which presupposes knowledge of the special conditions of parents with intellectual disabilities. Research on how new social workers experience the transition from education to practice reports that they do not always feel that their education has prepared them for the complexity they face in general social work practice (Tham and Lynch, 2021). In particular they report a lack of knowledge when they meet parents with intellectual disabilities (Lewis et al., 2015). Research on assessment methods to measure parental capacity highlights the importance of not only focusing on measurements using standardised instruments and scales, but the necessity to also use the accumulated experience-based knowledge of the social workers (Conley, 2003). Social workers point to a discrepancy between how the BBIC model as a standardised tool is supposed to work and how it is actually being used in their everyday practice (Skillmark and Oscarsson, 2020). Some research suggests that the use of standardised tools in social work practice might reduce social workers’ discretion and therefore negatively impact their ability to make professional judgements (Ponnert and Svensson, 2016; Skillmark and Oscarsson, 2020).

The concept of being a “good enough parent” has been highlighted as significant in research concerning assessments of the parental capacities in child protection cases and social work practices (Woodcock, 2003). The concept “good enough parent” suggests that one rejects the idea that there is such a thing as a “perfect parental ability” and instead focuses on what factors in the parental capacity that are sufficient to meet the child’s basic needs (Hoghughi and Speight, 1998). These factors can also be linked to the protective factors which are represented in the BBIC-model (Choate and Engstrom, 2014; Hoghughi and Speight, 1998).

Choate and Engstrom (2014) describe that in the context of assessing parental capacity in child protection investigations, it is important to note that ultimately a professional assessment is required. They describe that being a “good enough” parent is rarely clearly defined, but that it is still used as a factor in decision-making in the social work practice. One conclusion that Choate and Engstrom (2014) make is that more dialogue and research is required on how being a good parent plays a role in child protection investigations.

Previous research concerning child protection investigations shows that the parental capacity, particularly in parents with intellectual difficulties, is a complex task to assess (Ward et al., 2015; Lewis et al., 2015). It is necessary to consider both the Convention on the Rights of the Child to protect the development of children and young people, while at the same time protecting the rights of people with disabilities to be parents to their children, especially within the framework of risk and protection factor assessments. The concept of a “good enough parent” can make the assessment even more complex due to the undefined nature of the concept.

There is some research conducted on the experiences of the maternal role and support for mothers with cognitive limitations who have had their children placed in care (Janeslätt et al., 2019), as well as research on the experiences of mothers with intellectual disabilities having their children removed by the child protection services (Mayes and Llewellyn, 2012). There is also research conducted on the social workers’ experiences of working with parents with intellectual disabilities (Lewis et al., 2015), as well as research on the experiences of social workers perceptions of working with BBIC (Matscheck and Berg Eklundh, 2015; Rasmusson, 2004; Svendsen, 2012). Moreover, there is research conducted on parental capacity assessments in child protection services (Aunos and Pacheco, 2021; Curtis, 2009), but to the best of our knowledge is there a lack of research on social workers perceptions of assessing the parental capacity of parents with intellectual disabilities.

The social workers who are conducting the child protection investigations are tasked with judging whether parents (who are not always equipped to care for their own interests) have the capacity to care for their child. If the investigation is not managed properly, it can be seen as an infringement of the parents’ rights to pursue their pregnancy and child-rearing as stated by the UN convention. It is therefore of great importance to investigate how social workers who carry out child protection investigations perceive and experience the assessments of parental capacity of parents with intellectual disabilities.

The aim of this study was therefore to explore the social workers’ perceptions of assessing the parental capacity of parents with intellectual disabilities in child protection investigations. The following research questions were asked:

• How is the parental capacity of parents with intellectual disabilities assessed by social workers from child protection services?
• What challenges do social workers from child protection services experience when they are assessing the parental capacity of parents with intellectual disabilities?

## Method

### Research design and research method

This study adopted an explorative, inductive research design. Data were collected through four focus groups, each with three respondents. In two of the focus groups the respondents worked together and were known to each other. Respondents in the other two focus groups came from different workplaces. The focus group methodology was chosen because it allows for spontaneous interactions between respondents that can lead to a greater degree of reflexivity in the comments they make. The focus group method arguably gives respondents greater opportunities to clarify their views and articulate issues concerning a particular phenomenon, especially in comparison with one-to-one interviews (Kitzinger, 1995). The potential for a comparison of different views within a group can also lead to a greater breadth and depth of information as well as a greater insight into experiences (Carey and Asbury, 2012).

### Ethical consideration

The study was approved by an Ethics Committee of a Higher Education Institution in Sweden (2021-05-03).

Before recruiting participants, the managers of the respective social services departments were contacted in order to get permission and to ensure that the participation in the study would not add to the social workers’ workload as they were already considered to have a heavy workload. The participants were provided with oral and written information about the study and signed an informed consent form. In the information letter sent to the participants it was clearly stated that participation was completely voluntary, and they could withdraw from the study at any time, and this information was also repeated in connection with the focus group interviews.

In consideration of the emotions and thoughts that could have arisen during the focus groups, after the interviews the participants had an opportunity to remain in the video conference room and engage in group reflection on what had emerged during the interview.

### Recruitment process

Managers responsible for social workers within child protection in five social services offices in central Sweden were contacted to ask for approval to inform social workers about the study. After the approval was given, an information letter about the aim of the study, and the method as well as a consent form for participation was e-mailed to the managers. The managers then distributed the information letter and consent form to the child protection social workers. The social workers who volunteered to participate in the study later e-mailed the researcher who placed them into different focus groups. As each of the managers distributed the information letter and consent form in their own department it was impossible for the researcher to know how many of their employees had received but declined participation besides those who contacted the researcher and agreed to participate.

### Participants, setting and sample

Twelve professional social workers within social services in central Sweden took part in the four focus groups. The sample for the study was a non-random, purposeful sample. The inclusion criteria for participation were that the participants needed to have a Bachelor of Science in Social Work or equivalent relevant higher education in the field of social work, as well as more than six months’ work experience in the field of child protection prior to participation. Working in child protection services for longer than six months was considered sufficient work experience to be able to participate in the study because after six months, social workers were likely to have worked with parents with intellectual disabilities. Participant characteristics for each focus group are presented below.

*Focus group 1:* The age of the participating social workers ranged from 26-56 years and their work experience in assessing child protection investigations ranged from 4-11 years. The participants all had a Bachelor of Science in Social Work or equivalent education.

*Focus group 2:* The age of the participating social workers ranged from 32-42 years and their work experience in assessing child protection investigations ranged from 4-5 years. The participants had all a Bachelor of Science in Social Work.

*Focus group 3:* The age of the participating social workers ranged from 23-52 years and their work experience in assessing child protection investigations ranged from 1-7 years. The participants had all a Bachelor of Science in Social Work.

*Focus group 4:* The age of the participating social workers ranged from 23-29 years and their work experience in assessing child protection investigations ranged from 6 months-1 year. The participants had all a Bachelor of Science in Social Work.

### Data collection and analysis

The data was collected using the video conference platform Zoom to conduct the four focus group interviews, with three participants in each group. The focus group interviews were recorded in Zoom so the audio could be analysed. Prior to the interviews each participant received an e-mail containing a personal link to each video conference room. JN conducted all interviews. During the interviews a PowerPoint page containing questions of interest were shown to the respondents to help focus the discussions on specific themes. The interview questions concerned: Parental capacity and the assessments, meeting parents with intellectual disabilities, experiences of working with the parents and challenges. Each interview was one to two hours long with a total of six hours. The recordings were transcribed verbatim by JN.

The analyses of the transcribed data were conducted through an inductive, conventional content qualitative analysis (Elo and Kyngäs, 2008). First, the interviews were read thoroughly to give a sense of what all the focus groups expressed as common themes related to the aim of the study. In the open coding, the quotes from the social workers were condensed and the emerging codes were written in the margin of the transcript. The codes were then grouped together into broader condensed overarching categories describing common themes expressed by all the focus groups. These initially generated categories functioned as a way to structure the codes into different hierarchical and more broadly ordered headings where similar codes representing similar patterns or meanings could be grouped together. The codes under each grouping were later clustered into mutually exclusive categories and sub-categories as a mean to formulate descriptions of the main points of interest in the study. Finally, an overarching theme, according to Graneheim and Lundman (2004) was created, and the theme comprised the main point of the analysis. All coding, sub-categories and categories were discussed, developed and analysed together by both researchers until a consensus was reached.

## Results

One overarching theme, three main categories and ten sub-categories were created from the content analysis. The overarching theme was created out of the main findings taken from the categories. The summary of the results regarding the social workers’ perceptions of assessing the parental capacity of parents with intellectual disabilities is shown in Table 1. The results are presented with illustrative quotes taken directly from the transcribed data.

**Table 1.** Summary of results – Theme, Main Categories and Sub-categories

Theme		
**Parental capacity in relation to the child’s needs is assessed, not disability of parent**		
**Main categories**		
The process of assessing parental capacity	Changeable parental capacity	The social workers’ emotions and experiences in relation to the assessments
**Sub-categories**		
The role of intellectual disability in the assessments	The parental capacity dependence on time and place	The social workers’ emotions in the assessments
The assessment of risk, protection, and concerns	The impact of family and systems surrounding the child	The experiences of meeting parents with intellectual disabilities
Assessing parental capacity with assessment tools		Challenges of assessing parental capacity of parents with intellectual disabilities
Assessing ”Good enough”		

### Parental capacity in relation to the child’s needs is assessed, not disability of parent

One overarching theme was created when analysing the data. The social workers described that the parental capacity was always assessed in relation to each specific child’s needs, and even though the parents’ disability could have an impact on the parental capacity, the disability itself was of less importance during the child protection investigations.

### The process of assessing parental capacity

The social workers described different aspects and procedures when conducting their assessments of parental capacity of parents with intellectual disabilities. The procedure of assessing parental capacity was made using both investigatory procedures and the use of various risk assessment tools available to the social workers in their practice.

#### The role of intellectual disability in the assessments

The social workers described how the intellectual disability could have an impact when assessing the overall situation of the family. At the same time, if the assessment was based solely on the parental capacity of the parents, the intellectual disability itself was not in focus.

*“I would like to say that the intellectual disability plays a role, but perhaps more in how we treat those parents, work with those parents than in our assessment, because it is still the capacity to care, linked to the child’s needs that is being assessed.” – (Focus group 2)*

The social workers also described how the assessment of all parents was assessed in the same way, regardless of impairment. They also made clear that the children’s needs and the parental capacity to meet those needs were the focus of the assessment.

*“If the child is being harmed and the parent does not want to, or cannot change, then of course it matters, but intellectual disability does not matter more than any other impairment or inability or drug abuse or illness.” – (Focus group 1)*

When reflecting on the parental capacity of parents with intellectual disabilities, the social workers explained that both research and other available resources within their field of practice depicted that parents with intellectual disabilities may find it more difficult to care for their child due to the intellectual disability.

*“…there is research that shows that it almost always turns out that if you have been diagnosed with intellectual disability, you have such great difficulties that it is difficult to be a parent…”; “In our manuals about BBIC it says… that it is a risk if the parent has an impairment that really affects the parenting ability.” – (Focus group 1)*

The social workers perceived that intellectual disability could have an impact on the parent’s ability to give a basic care for their child, but it is not *per se* the most important aspect in the assessments.

*“…it’s about how well the parent are able to be a parent, of course it [intellectual disability] matters…” – (Focus group 2)*

*“To have an intellectual disability does not per se make me assess the parental capacity as lower but the entirety of the child’s life, and the child’s needs.”; “It depends on the severity of intellectual disability, is it a very great, which affects the child very much, then yes, it absolutely matters.” – (Focus group 4)*

The social workers also described that the parental capacity could be seen as a facilitator of the child’s attachment needs where the intellectual disability could have a great impact on the child’s own attachment, which in turn can weaken the parent’s own parental capacity.

*“I am thinking of a case where a girl who has had great attachment difficulties due to how her mother was and it meant she had very high demands on a parent’s parenting ability, so somehow it is the mother’s disability that has created higher demands on herself that she cannot possibly meet.” – (Focus group 1)*

#### The assessment of risk, protection, and concerns

The most important aspects when assessing parental capacity were the assessment of various risk and protection factors in families. By assessing them in relation to the concerns that have been reported to social services the social workers could determine how these factors might affect the children. The parents were judged to lack parental capacity if the risk factors outweighed the protective factors, and at the same time the concerns for the child’s safety or health are so serious that the parental capacity could not be considered as good enough.

*“We map out: What are the concerns, what protection is there, what needs to be done, are there complicating factors in this… then you can draw conclusions about what the risks are, what protection there is based on what we know. And it is not always that we really know how it really is, we have to assess based on what we receive [in the investigation].” – (Focus group 2)*

*“We look for consequences for the children on the parent’s abilities: What consequences do the parents’ behaviours have on the child, is it something that we see that causes concerns, is it something that harms the children or does not help them develop…” – (Focus group 1)*

The capacity could not be measured in an objective way using rating scales because each parent’s parenting capacity was related to the uniqueness of each individual case regarding the consequences.

*“It is impossible to grade and measure [the parental capacity]…. but I think you need to measure it against the child's needs.” - (Focus group 1)*

*“There is like no measurement scale that you can just check off: these parts [of the parental capacity] are worth 10 points.” – (Focus group 2)*

#### Assessing parental capacity with assessment tools

The social workers highlighted the use of various assessment resources at their disposal to assist them during the child protection investigations and the parental capacity investigations. They described the use of both the assessment tools, BBIC and Signs of Safety, to assist in the assessment of risk and protection factors, as well as for the assessment of the initial concerns of the families.

*“We use BBIC when we do our assessments which is pretty good because you can use it as a template to follow, what is it that you need to achieve; and the parenting ability versus the child's needs and vice versa as well.” – (Focus group 3)*

*“We work a lot with Signs of Safety, when making the assessments, and then it is, what are the concerns and what works and what is good, and it is there where you often find out what the risks are.” – (Focus group 2)*

*“I think that it is through these different aspects described by BBIC, which you follow according to the model – these are based on what BBIC has said parental capacity must contain;” “…but you don’t always write something about each and every aspect if it is not about the main concern.” – (Focus group 4)*

The social workers also described the use of investigation homes, home investigations and family therapists when conducting investigations and assessments of parental capacity. They also highlighted the importance of the experience-based knowledge of their colleagues when assessing the parental capacity in each case.

*“Often when we have parents with disabilities, intellectual disabilities, it can be impossible for us to see from just a few visits, so then we need to place them in investigation homes to observe how they are managing (behaving) during both day and night. And it can also, it can be something that is good because then you get to see what the parental capacity looks like…” – (Focus group 1)*

*“We are indeed making these assessments [of parental capacity], but it is a collaborative process to be able to capture all these different aspects. This is because all [colleagues] have different personal and professional experiences of different cases.” – (Focus group 4)*

#### Assessing “Good enough”

The social workers pinpointed a “gatekeeping” function called *good enough parenting* linked to a parent’s parental capacity to care for their child’s needs. If the parent managed to care for the needs of their child, then the parental capacity could be assessed as good enough. The social workers described that the threshold of being good enough is when the protective factors were greater than the risk of harm or developmental problems for the child.

*“Parental capacity is always sort of linked to if it is good enough, so to speak, it is linked to the children's needs.” – (Focus group 1)*

*“Good enough it is like a bit like that, when you have to make a choice about what will be the least harmful [for the children]”; “We look at what we are worried about and what risks are present, what kind of protection there is. If the protection factors outweigh the risks then maybe it’s good enough and we do not have to do anything about it, if not: what do they need to be able to reach that level.” – (Focus group 2)*

The social workers also described situations where a parent’s parental capacity could be judged as good enough, even though they felt worried or clearly could see that the whole living situation around the child was not entirely satisfactory. In those cases, they may lack the authority to intervene in the family using coercive legislations.

*“Well, you can have a little stomach ache, or, well maybe, yes I am a little worried – but it works, it is okey as it is. It is enough, it meets his [the youth] needs.” – (Focus group 4)*

*“We saw that it was not completely fine at home, it was dirty, filthy … it was not good, but she [the parent] did not want any interventions, and there were not enough reasons to use coercive legislation, so you had to move on. She was judged to be good enough….” – (Focus group 1)*

### Changeable parental capacity

The social workers described parental capacity as a phenomenon that was not only linked to being a parent alone, but to the whole situation regarding the parent’s own ability to care for their child. They expressed that the capacity could change as the child’s own individual needs changed, or the parent’s attitude or willingness to change their behaviour changed. The social workers also expressed a time dimension of parental capacity regarding both the child and the parent. This means that many things can happen over time, such as, the changing needs of the child, as well as the support structures surrounding the family and their ability to help them.

#### The parental capacity dependence on time and space

The social workers explained that the parental capacity was linked to factors in both the present and the future, and that the capacity could change depending on situational changes in a time and space continuum.

The changes could be affected by the parent’s ability to cope with factors linked to their own disability, life-situation, and predisposition to change, as well as the child’s own development and specific age-related needs at different stages of life.

*“You assess the parenting abilities a little differently depending on the age of the child, as they have different needs; an infant needs more of the basic needs met, as well as closeness, food, care, while a school child’s needs, are on many more levels.” – (Focus group 2)*

*“The parental ability can absolutely change; it all depends on receptivity, insight maybe, and then I do not say that everyone has that ability, but you still have to try to focus on the possibilities [for change].” – (Focus group 3)*

The social workers pointed out that a child’s individual needs were an important factor when trying to pinpoint a parental capacity. They described that the parental capacity was always recognized and judged in relation to the needs and consequences of a particular child. The parents could be a satisfactory parent for one child but not for another.

*“It is always about the child's needs; always, always, always, but first you must figure out what this child needs; why did they end up here [at the social services] and what can we see that we can help with, can we or the parent compensate for this in some way.” – (Focus group 3)*

*“It is quite a fluid concept [parental capacity], but in some way is it about satisfying the needs of a particular child.” – (Focus group 4)*

#### The impact of family and systems surrounding the child

The social workers described the impact of the surrounding network and family when parental capacity was being determined. They reasoned that the immediate family, such as another parent, a grandparent or even a neighbour could compensate the lack of parental capacity in the families.

*“There might be another parent who is involved and supports [the families] in troublesome areas, and then they might still manage to do well.” – (Focus group 2)*

The social workers underscored the importance of professional systems such as schools, agencies such as social welfare, and family therapists as a mean to support families and compensate limited parental capacity.

*“They have been in close contact with habilitation services for persons with disabilities. They [the family] are used to receiving support, it is both good and a bad thing, but, in this case with the one-year-old, the professional network has been able to help these parents stay one step ahead in development. In the child’s development.” – (Focus group 3)*

The social workers also expressed that family networks were an important facilitator of protection and safeguarding for children who might become victims of harm due to limited parental capacity. At the same time, especially in families where matters of honour were present the family network can instead function as a facilitator of harm.

*“Having a network is often a protection in itself, but there are exceptions such as in honour cases where the network is also what makes it a risk…” – (Focus group 2)*

### The social workers’ emotions and experiences in relation to the assessments

The social workers’ perceptions of working with parents with intellectual disabilities in child protections investigations generated three sub-categories. The social workers described both the difficulties and emotional aspects of the assessments linked to the professional role as an assessor, as well as the experiences of working with parents which were not always fully aware of or acknowledged their disability.

#### The social workers’ emotions in the assessments

The social workers described the emotions they felt when trying to assess the parental capacity of parents with intellectual disabilities. Most of the emotions were linked to the difficulties of making the assessment of what being a “good enough parent” was in relation to the intellectual disabilities of the parents. The social workers expressed feelings of frustration and unease during the assessment of good enough parenting because of the difficulties of assessing which aspects of the capacity were decisive for the best interests of the child. But also, because they were sometimes forced by legislation and regulations to judge a parental capacity as good enough even though in their professional role they did not always feel that it was good enough for the child.

*“Good enough … those are the matters that are the most difficult in our job, and when you end up in a situation that: Yes, it might as well be good enough, although you do not feel that it is good at all, but it is good enough. That’s when you toss and turn in bed and lie awake at night”; “It is the worst expression there is in social services. I really hate good enough.” – (Focus group 1)*

*“What is worst for the child… least bad: To be without love or to have rubber boots. Who are we to sit and decide? You often end up with that, who are we to determine this child’s life.” – (Focus group 2)*

#### The experiences of meeting parents with intellectual disabilities in the practice

The social workers described the experiences of meeting parents with intellectual disabilities. They talked about the fact that the most difficult part was the uncertainty of whether or not the parent had a disability or not. They described that the uncertainty made it harder to conduct a good investigative interview as they could not be sure that the parents were aware of the ongoing investigation and why their parental capacity was investigated. The social workers described that it was hard to know if the parents had the ability to comprehend and receive the information that the social services were trying to present to them. They also related that it would be easier if they knew of the parent’s intellectual disabilities during the meetings. When the disability was known, the social workers could adapt the communication and information to the parent’s abilities to understand.

*“Can I adjust my practice based on the needs of the person in front of me … and just try to do my best to meet this person as good as possible, then it can actually turn into something good.” – (Focus group 3)*

*“It is even more important [to use a simple language] with parents with disabilities so that you communicate with them on their level, so they can understand the situation and our concerns about their children.” – (Focus group 2)*

The social workers said that parents that might have an intellectual disability often tried to hide the fact that they had an intellectual disability by the use of techniques such as: the use of quotes and phrases rehearsed beforehand that they thought the social workers wanted to hear; or by trying to hide their shortcomings by referencing other diagnoses they might have other than an intellectual disability. The social workers also said that some parents had learned how to act in the meetings so as to appear as though they did not lack parental capacity.

*“You [the parents] can learn what to say; you have your 5 phrases that you know work when you want to make people nearby happy and calm.” – (Focus group 1)*

*“But sometimes you can learn what to say, how to behave and what must work, seen from the outside. Then you are “smart” in that way and appear to have no disability outwardly at least.” – (Focus group 3)*

#### Challenges of assessing the parental capacity of parents with intellectual disabilities

The social workers described challenges when it came to assessing the parental capacity of the parents. They described that deciding what is good enough was a challenge because of the uncertainty of the future development of the child. During initial assessments, the social workers could see that the capacity was good enough right now, but that it might not be good enough at a later stage. The difficulty of considering and assessing the needs over the long term made the assessments difficult as the social workers had to take the future risk of harm in consideration during the present assessments. The social workers also described difficulties in assessing the whole situation for the family when they could see that the family had many protection factors, but still lacked the capacity to meet all the child’s needs.

*“I think there is a big challenge regarding this “good enough”; what is good enough, with these particular parents”; “You have to think about this future risk, what does it mean if these aspects are very non-functional but there are 4-5 other aspects that actually work very well.” – (Focus group 2)*

The social workers also described that one major challenge when assessing the parental capacity was that of not knowing if the parent actually had an intellectual disability or not, due to them hiding their potential disability. The social workers explained that attempting to hide a disability might be due to feelings of shame and the fear of being stigmatized as a parent with an intellectual disability. The social workers expressed that the parents often tried to avoid “telling the truth” about a potential diagnosis because of this stigmatization, which in turn made it more difficult for the social workers to assess the parental capacity of the parents.

*“No one is so open-minded that they would say that they have it [intellectual disability], and that they are fine with it.” – (Focus group 1)*

*“I think you can understand the shortcomings in the parental capacity more easily if there is a pronounced disability, otherwise it is more difficult to put your finger on what it is that makes it not good enough.” – (Focus group 2)*

## Discussion

The aim of this study was to explore social workers’ perceptions of assessing parental capacity of parents with intellectual disabilities in child protection investigations. Two research questions were asked concerning how the parental capacity was assessed and what challenges social workers experienced when making these assessments.

One main finding of this study was that both the disability and the parental capacity were considered in the assessments, but that the main focus was on the capacity not the disability. Parents with intellectual disabilities were described as finding it more difficult to be a “good enough parent” due to their disability, while at the same time when the parental capacity was assessed the intellectual disability of the parent was not in focus. Research on assessments of parental capacity of mothers with intellectual disabilities have found that child protection social workers tend to embed bias and stereotypes linked to the intellectual disability when making their assessments (Aunos and Pacheco, 2021). The expressed focus on the assessment of capacity in this study, without taking into account the parents’ intellectual disability during the assessments, may suggest a contradiction between our results and those of Aunos and Pacheco (2021). The social workers in the present study also expressed that when working according to the BBIC procedures, the guidelines indicate that the intellectual disability of the parent can be viewed as a risk factor in itself. This perception of the disability as a risk factor is also observed by several other previous studies focusing on the discrimination and injustice of parents with intellectual disabilities in child protection services (Callow et al., 2016; Sigurjónsdóttir and Rice, 2017, 2018). Overall, our finding could imply a discrepancy between what factors the social workers think they take into consideration when making their assessments, and the awareness of a potential risk of bias of making assumptions based on the disability of the parent. To recognise and acknowledge this discrepancy can be of great importance when developing future practice in child protection services.

As previous research has shown, parental capacity is often linked to risk and protection factors in the family’s potential to provide basic care for their child (Emerson and Brigham, 2013; Feldman et al., 2012; Lindberg et al., 2017; McGaw et al., 2010; Sidebotham and Heron, 2006). Having an intellectual disability is often more likely to result in difficulties concerning economical, psychosocial, and emotional factors in families, and this may impact negatively on children (Ahonen et al., 2015; Appleyard et al., 2005; Feldman et al., 2012; Loeber et al., 2009). The results of the present study indicate that these social workers, who are employed to assess what (if any) interventions are needed to support the families, are aware of what difficulties the parents face when being assessed by the child protections services. The social workers not only comprehend the difficulties of facing stigmatisation and shame linked to the intellectual disabilities, but they also showed knowledge of the difficulties the parents might have when trying to understand the procedures of being assessed during a child protection investigation. These findings confirms the earlier findings of Lewis et al. (2015) who found a perceived power imbalance expressed by the social workers, between the social workers and parents. This power imbalance is observed through the fear the parents exhibit of being exposed as having an intellectual disability, as well as their use of techniques and behaviour to hide it. The knowledge about a perceived power imbalance and how it can be expressed through the parents attempts to hide the disability is an important connection to make, especially as the social workers in the present study expressed the importance of knowing about the disability to be able to do a professional job during the investigations. By helping the parents to cope with stigmatisation, and offering support, the social workers might be able to reduce the power-imbalances perceived by both the social workers and the parent.

The present study also highlights the difficult-to-assess situations that social workers within child protection services faced in their practice. The need for making assessments based on risk and protection factors is bound to a temporal dimension, in which both present and future factors and outcomes must be considered in the assessments made in the present. Making these assessments was perceived as one of the main challenges for the social workers when conducting the parental capacity assessments. The work of Choate and Engstrom (2014) highlights the difficulties in assessing what a “good enough parent” is in relation to future risks and negative outcomes. The present study underscores the challenges experienced by social workers suggesting that they might be linked to the difficulties of finding the threshold of when a parental capacity is good enough. The concept of the “good enough parent” and the illusive nature of where to draw the line for what good enough is, is experienced as the most difficult challenge to handle both professionally and emotionally by the social workers. This suggests an inherent ethical dilemma for the practice of making parental capacity assessments as part of the child protection investigations.

### Methodological considerations

The judgement of trustworthiness in this study has been based on the definitions of credibility, dependability, transferability and confirmability in qualitative research taken from Lincoln and Guba (1985). The credibility and transferability of this study could be considered to be quite high because all of the focus groups were engaged and had similar discussions about the research topics presented to them. The participants shared their knowledge and thoughts generously and the interview questions were posed as openly as possible, which may have contributed to the rich descriptions that were collected. The use of focus groups could also be considered to have had a positive impact on the quality of the data because the group interactions made it possible to collect a great breadth and depth of information (Carey and Asbury, 2012). It is possible that biases linked to conformism and other cognitive mechanisms might have hindered the collection of each respondents’ individual views on a phenomenon (Acocella, 2012), which is important to have in mind when interpreting the results of this study. The inductive design of the study could also be considered to have contributed to its higher confirmability because the findings have stayed close to the collected data. One limitation is the rather low number of participants due to the Covid-19 pandemic. As the main findings of this study are not linked to any specifically Swedish legislation or policies, it is possible that the findings may apply to similar settings or contexts. To establish credibility and confirmability, reflexivity was used throughout the research process, with both researchers collaborating in data analysis and negotiating the outcome. Furthermore, five social workers who took part in the study responded to the distribution of a written respondent validation.

### Conclusions

An intellectual disability can affect both the assessment of parental capacity, as well as the investigatory processes of social workers. The study shows that the children’s needs and the parental capacity to meet those needs are the focus of the assessment, not the parent’s disability. Key factors that can help to reduce the power imbalance between social worker and parent are adapting communication used and offering support. The study shows that it is a complex challenge for the social workers to assess a future risk of harm and neglect using investigations done in the present, and thus, more research is needed on how the future risks can be assessed. Furthermore, the study shows that assessing what is good enough parenting is viewed as the most difficult task when conducting the parental capacity assessments in relation to parents with intellectual disabilities.
